# ABC transporters as multidrug resistance mechanisms and the development of chemosensitizers for their reversal

**DOI:** 10.1186/1475-2867-5-30

**Published:** 2005-10-04

**Authors:** Cheol-Hee Choi

**Affiliations:** 1 Research Center for Resistant Cells, Chosun University Medical School, 375 Seosuk-dong, Dong-gu, Gwangju 501-759, South Korea

**Keywords:** ABC transporter, bioavailability, chemosensitizer, drug resistance, P-glycoprotein, multidrug resistance associated protein, breast cancer resistance protein.

## Abstract

One of the major problems related with anticancer chemotherapy is resistance against anticancer drugs. The ATP-binding cassette (ABC) transporters are a family of transporter proteins that are responsible for drug resistance and a low bioavailability of drugs by pumping a variety of drugs out cells at the expense of ATP hydrolysis. One strategy for reversal of the resistance of tumor cells expressing ABC transporters is combined use of anticancer drugs with chemosensitizers. In this review, the physiological functions and structures of ABC transporters, and the development of chemosensitizers are described focusing on well-known proteins including P-glycoprotein, multidrug resistance associated protein, and breast cancer resistance protein.

## Background

**Figure 1:**
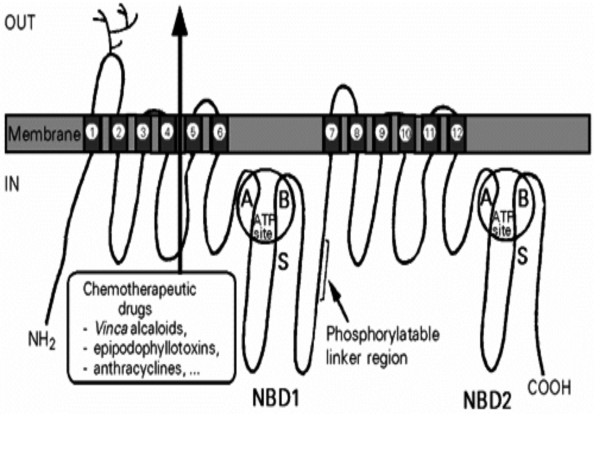
Schematic structural organization of P-glycoprotein. Each half contains a highly hydrophobic domain with 6 transmembrane α-helices involved in chemotherapeutic drug efflux, and a hydrophilic domain located at the cytoplasmic face of the membrane, nucleotide binding domain 1(NBD1) or NMD 2, containing an ATP-binding site with cheracteristic Walker motifs A and B and the S signature of ABC transporters. The two half molecules are separated by a highly charged "linker region which is phosphorylated at several sites by protein kinase C and the first extracellular loop is heavily *N*-glycosylated [3].

One of the major problems related with anticancer chemotherapy is resistance against anticancer drugs. Some cancers such as non-small cancer, lung cancer, and rectal cancer show what is called primary resistance or natural resistance in which they do not respond to standard chemotherapy drugs from the beginning. On the other hand, many types of sensitive tumors respond well to chemotherapy drugs in the beginning but show acquired resistance later. Experimentally, drug resistance could be very specific to the drug used due to abnormal genetic machinery such as gene amplification within tumor cells in many cases. Multidrug resistance (MDR) is especially problematic in acquired drug resistance. MDR is the phenomenon in which cancer cells exposed to one anticancer drug show resistance to various anticancer drugs that are structurally and functionally different from the initial anticancer drug. The most investigated mechanisms with known clinical significance are: a) activation of transmembrane proteins effluxing different chemical substances from the cells; b) activation of the enzymes of the glutathione detoxification system; c) alterations of the genes and the proteins involved into the control of apoptosis (especially p53 and Bcl-2). The cell membrane, cytoplasm, and nuclear protein participate in these resistance mechanisms [1]. The resistance mechanism is called typical MDR or classical MDR when overexpression of the membrane efflux pumps is involved in MDR. The classical MDR is due mostly to increased efflux pumps in the cell membrane of cells pumping anticancer drugs out of cells. The most typical efflux pumps in the cell membrane is P-glycoprotein (Pgp) [2] having the molecular weight of 170 KD, due to the gene amplification of the normal human gene, *MDR1*. The efflux pump Pgp is responsible for transporting various xenobiotics (not limited to anticancer drugs) out of cells by using ATP (Fig. 1) [3]. Pgp is one of the membrane transporter superfamily having the ATP-binding cassette (ABC) with well-preserved homology of the site where ATP binds. There are more than 100 ABC transporters distributed from prokaryotes to humans. Forty-eight ABC genes have been reported in humans, among which the functions of 16 genes have been determined and 14 genes are related with diseases present in humans (cystic fibrosis, adrenoleukodystrophy, Stargardt's disease, drug-resistant tumors, Dubin-Johnson syndrome, Byler's disease, progressive familiar intrahepatic cholestasis, X-linked sideroblastic anemia, ataxia, and persistent and hyperinsulimenic hypoglycemia in children) [4,5].

Other efflux pumps of the mammalian cell membrane in ABC superfamily include multidrug resistance-associated proteins (MRP) [6] and breast cancer resistance proteins (BCRP; mitoxantrone resistance proteins, MXR) [7,8]. Other than the fact that these resistant proteins belong to the ABC superfamily, they are quite different with respect to gene locus, amino acid sequence, structure and substrate (Table 1 and 2). In this review, the physiological functions and structures of ABC transporters, and development of chemosensitizers are described focusing on well-known proteins including Pgp, MRP, and BCRP.

**Table 1:** Gene locus and tissue distribution of ABC transporters

**Name**	**Alternate name**	**Gene locus**	**Tissue distribution**
MDR1	ABCB1, P-GP	7q36 [9]	Gut (apical membrane), liver (canalicular membrane), kindey (apical membrane of epithelial cells of proximal tubule), blood brain barrier (luminal membrane of endothelial cells), testis (endothelial cells of capillary), placenta (trophoblast)
MRP1	ABCC1	16p13.1 [6]	Many tissues (brain etc)
MRP2	ABCC2, cMOAT	10q24 [10]	Liver, gut, kidney, placenta
MRP3	ABCC3	17q21.3 [11]	Liver, gut, adrenal cortex, placenta
MRP4	ABCC4	13q32 [11]	Many tissues
MRP5	ABCC5	3q27 [11]	Many tissues(brain etc)
MRP6	ABCC6	16p13.1 [12]	Liver, kidney
MRP7	ABCC10	6p12-21 [13]	Many tissues
MRP8	ABCC11	16q12.1 [14]	Breast, testes
BCRP	ABCG2, MXR1, ABCP	4q22 [15]	Placenta (syncytiotrophoblasts), intestine (epithelium), liver (canalicular membrane), breast (ducts and lobules), endometrium (vein and capillary but not artery), gut

**Table 2:** Endogenous and exogenous substrates for ABC transporters

**Name**	**Endogenous substrate**	**Exogenous cytotoxic substance**
MDR1	Estrogen glucuronide conjugates (estradiol, estriol), endorphin, glutamate, steroids (cortisol, aldosterone, corticosterone), beta-amyloid, 1-O-alkyl-2-acetyl-sn-glycero-3-phosphocholine (generically platelet-activating factor, PAF)	Anthracyclines (doxorubucin, daunorubicin, epirubicin), actinomycin D, colchicine, podophyllotoxin (etoposide, teniposide), methotrexate (only in carrier-deficient cells), mitomycin C, mitoxantrone, taxenes (paclitaxel, docetaxel), vinca alkaloids (vincristine, vinblastine)
MRP1	Estradiol-17beta(beta-D-glucuronide) glutathione, glutathione S-conjugate leukoetriene C4, glucuronosyl bilirubin	Anthracyclines, cochicine, etoposide, heavy metals (arsenite, arsenate, antimonials), vincristine, vinblastine, paclitaxel
MRP2	Estradiol-17beta(beta-D-glucuronide), glutathione, glutathione S-conjugate Leukoetriene C4, glucuronosyl bilirubin,	Cisplatin, CPT-11, doxorubicin, etoposide, methotrexate, SN-38, vincristine, vinblastine
MRP3	S-(2,4-dinitrophenyl)glutathione	Cisplatin, doxorubicin, etoposide, methotrexate, teniopside, vincristine,
MRP4	Glucuronide and glutathione conjugates	Methotrexate, nucleotide analogs, PMEA*
MRP5	Glutamate and phosphate conjugates	Doxorubicin, methotrexate, nucleotide analogs, topotecan,
MRP6	Cyclic nucleotides (cAMP, cGMP), glutathione conjugate	Doxorubicin, etoposide, teniposide
MRP7	?	?
MRP8	17beta-estradiol-(17-beta-D-glucuronide), leukotriene C4, cyclic nucleotides	5'-Fluorouracil, 5'-fluoro-2'-deoxyuridine, 5'-fluoro-5'-deoxyuridine, PMEA*
BCRP	Heme or porphyrin	Anthracyclines, bisantrene, camptothecin, epirubicin, flavopiridol, mitoxantrone, S-38, topotecan

* PMEA, 2',3'-dideoxycytidine 9'-(2'-hosphonylmethoxynyl)adenine

### Functions of ABC transporters

Although the physiologic functions of ABC transporters are not well known, they are expressed constitutively in not only tumor cells but also normal cells in the digestive system including the small intestine, large intestine, liver, and pancreas; epithelial cells in the kidneys, adrenals, brain, and testes; and endothelial cells (Table 1). From the aspect of the tissue distribution, ABC transporters are thought to participate in the absorption and secretion of endogenous and exogenous substances. Endogenous and exogenous substrates for ABC transporters reported so far are summarized in Table 2. Especially, the ABC transporters have shown to function as an efflux pump for lipid, multiple drugs, natural products and peptides. It is proposed to operate as a hydrophobic vacuum cleaner, expelling non-polar compounds from the membrane bilayer to the exterior, driven by the energy of ATP hydrolysis [143]. ATP-dependent transbilayer lipid transporters are classified into cytofacially-directed flippases and exofacially-directed floppases. Floppase activity has been associated with the ABC transporters although not all ABC transporters are floppases [144]. Endogenous substrates for Pgp include corticosterone [145], beta-estradiol 17beta-D-glucuronide, an endogenous cholestatic metabolite of estradiol [146], 1-O-alkyl-2-acetyl-sn-glycero-3-phosphocholine (generically platelet-activating factor, PAF) [147], glutamate [148] and endorphin [149]. It was also recently reported that Pgp has the function of removing beta-amyloid, which was reported as the causal substance of Alzheimer's disease [150,151]. MRP1 effluxes various conjugated substrates such as leukotriene C4 conjugates [152], steroid conjugates [153] and the GSH conjugate of aflatoxin B1, which is a mycotoxin [154]. Cells can, upon hypoxic demand, use BCRP to reduce heme or porphyrin accumulation, which can be detrimental to cells [155]. When cancer originates not only from cells normally expressing efflux pump but also cells having genes but not expressing, gene expression is initiated due to the exposure to anticancer drugs, resulting in resistance to anticancer drugs, eventually interfering with chemotherapy.

Pgp is mainly present in the apical membrane of intestinal mucosal membrane and lowers bioavailability of drugs by preventing the absorption of the drugs. Digoxin, which shows a low bioavailability and is mainly excreted through stool in normal mice due to poor absorption in the mouse intestine, shows a high bioavailability and mainly excreted through urine in mice with *mdr1 *knocked out[156]. The bioavailability of the substrate of Pgp, paclitaxel, also increased significantly in mice with *mdr1 *knocked out and in mice administered with the Pgp inhibitor, PSC-833 [157].

Recently, multiple *MDR1 *polymorphisms including more than 20 single nucleotide polymorphism (SNP) have been identified. The mutations at positions 2677(G→T) and 2995(G →A) of *MDR1 *in normal cells were firstly reported [158]. *MDR1 *polymorphism could be not only associated with alteration of Pgp expression and/or function, drug disposition and treatment outcome but also increase the risk of diseases such as Parkinson's disease and ulcerative colitis [159]. The influence of *MDR1 *SNP(C3435T and G2677T) on disposition of Pgp substrates or treatment outcome has been examplified in digoxin, phenytoin, fexofenadine, nelfinarvir, cyclosporine, talinolol and loperamide [159]. Polymorphisms of other ABC transporters have been reported [160-163].

If a substance in food affects Pgp, this substance also could affect the bioavailability of substrate drugs for Pgp. It was reported that substances present in grape juice or orange juice could increase the bioavailability of a drug being the substrate of Pgp by inhibiting it. [164]. These substances could also affect pharmacokinetics of other drugs [160,165]. On the contrary, some drugs could increase the expression of Pgp. St John's Wort used as an antidepressant increases the expression of Pgp, so it could significantly lower the serum concentration of indinavir or cyclosporin [166]. Digoxin is the substrate of Pgp and induces paclitaxel resistance by increasing Pgp [166]. Not only Pgp but also MRP and BCRP could affect the bioavailability of drugs.

One of the important physiological functions of efflux pump present in the cell membrane is to provide a pharmacological sanctuary for tissues present in the blood-tissue barriers such as in the case of blood-brain barrier (BBB), blood-placental barrier and blood-testes barrier. Hydrophilic substances present in blood could not go into tissues when they are not small enough to pass through the tight junction with simple diffusion. Nonetheless, various hydrophobic substances could not enter these tissues because they are effluxed out by efflux pumps. Actually, Pgp effluxes neurotransmitters or neuromodulators such as glutamate [148] and opioids [149,167] into blood from the brain. Compared with wild-type mice, drugs beings the substrate of Pgp were significantly increased in the brain of fetus when the *mdr1 *gene is knocked out in mice [168-171]. When the BCRP inhibitor, GF120918, was introduced to pregnant mice, the topotecan level was increased by two-folds in mouse fetus, suggesting that BCRP would function as the maternal-fetal barrier in the placenta [172]. Thus, quantitative and qualitative changes of transporters present in the membrane could affect pharmacokinetics such as the distribution of endogenous and exogenous substances.

### Structure of ABC transporters

**Figure 2:**
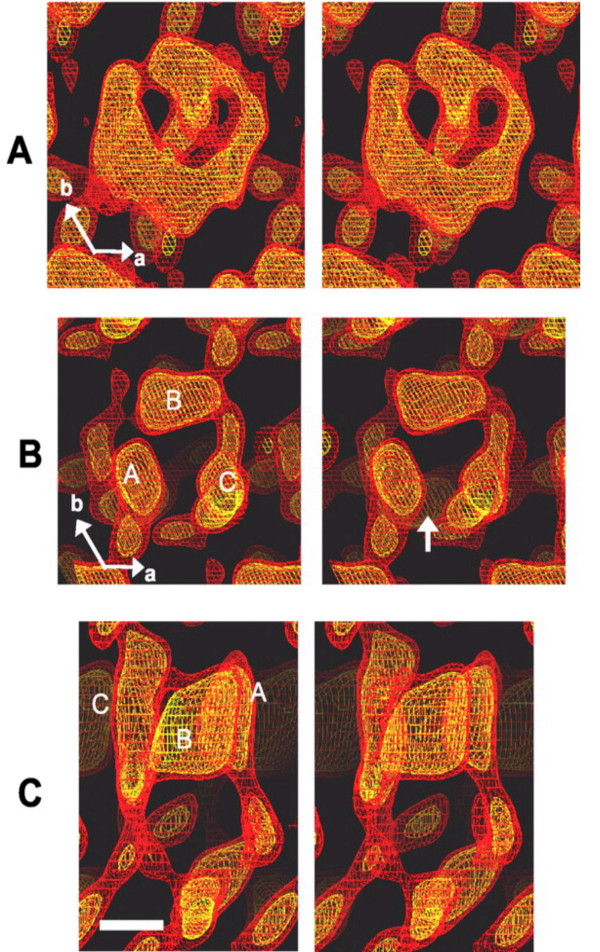
Comparison of nucleotide free-Pgp (nf-Pgp) and Pgp-AMP-PNP (Pgp-AMP-PNP) three-dimensional structures. *A*, stereo pair of the nf-Pgp three-dimensional structure, displayed using netting at 1.0 σ (*red*) and 1.5 σ (*yellow*) above the mean density level and viewed perpendicular to the crystal plane from the more heavily stained side (corresponding to the extracellular surface). *B*, equivalent views of the Pgp-AMP-PNP structure. The *arrow *indicates the gap along one side of the central pore. The locations of the three discrete densities *A*, *B*, and *C *are indicated. *C*, stereo pair of a side view of Pgp-AMP-PNP with the same color scheme as above. The directions of the principle crystallographic axes *a *and *b *are shown. *Scale bar *= 2.2 nm. AMP-PNP, non-hydralizable ATP analogue [176].

Pgp is a 170-kDa membrane protein glycosylated at the first extracellular loop (Fig. 1). Pgp is composed of 12 hydrophobic transmembrane domains (TMDs) and 2 nucleotide-binding domain (NBD). One NBD connects two TMDs with a hydrophilic NBD loop. TDMs form channels for substrate drugs, determine the characteristics of substrate, and efflux substrate drugs whereas NBDs are located in the interior of cytoplasm, and participate in ATP binding and hydrolysis [173]. Pgp undergoes conformational changes upon binding of nucleotide to the NBDs [174]. Rosenberg et al. have analyzed the three-dimensional structures of Pgp and its conformational change in the presence and absence of nucleotide [175-177]. The projection of the protein perpendicular to the membrane is roughly rectangular with a maximum depth of 8 nm, a pore size of 2.5 nm and two 3-nm lobes exposed at the cytoplasmic face of the membrane. The conformational change revealed a major reorganization of the TMDs throughout the entire depth of the membrane upon binding of nucleotide (Fig. 2A). In the absence of nucleotide, the two TMDs form a single barrel 5–6 nm in diameter and about 5 nm deep with a central pore that is open to the extracellular surface and spans much of the membrane depth. Upon binding nucleotide, the TMDs reorganize into three compact domains that are each 2–3 nm in diameter and 5–6 nm deep (Fig. 2B). This reorganization opens the central pore along its length in a manner that could allow access of hydrophobic drugs (transport substrates) directly from the lipid bilayer to the central pore of the transporter [176].

**Figure 3:**
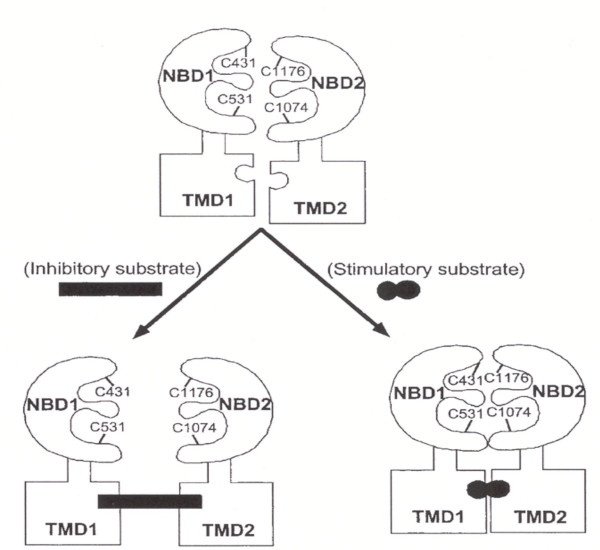
Model of the NBD conformational change by the drug binding to TDM. [178].

When one of two NBDs of Pgp is inactivated, not only drug transport but also ATP hydrolysis of normal NBD is inhibited. This result indicates that two NBDs would function cooperatively and they could not hydrolyze ATP independently [178]. It was recently reported that structural changes of NBDs are brought about when a drug binds to TMD so that the distance between NBDs is changed to affect the activity of ATPase as shown in Fig. 3[179]. Unlike in Pgp, however, the substrate leukotriene C4 could not be transported once NBD2 is inactivated but the substrate transport could not be inhibited when NBD1 is inactivated in MRP1 [180]. This result suggests that among ABC transporters, interactions of NBDs are not simple but function differently for every transport. Although the exact site and number of Pgp binding with drugs have not yet been determined, the important binding sites such as TMD 4, 5, 6, 10, 11 and 12 have been determined [181] whereas substrate drugs do not bind to NBDs [182].

### Development of chemosensitizers to overcome resistance

One of the causes for the failure of chemotherapy in the treatment of cancer is the emergence of MDR. Once MDR appears, chemotherapy is not effective even when using high doses of drugs enough to overcome resistance, toxic effects are brought about and the resistance mechanism could be further stimulated. These problems could be resolved by the use of the anticancer drugs that could bypass the resistance mechanism. For example, we could use other anticancer drugs such as alkylating drugs (cyclophosphamide), antimetabolites (5-fluorouracil), and the anthracycline modified drugs (annamycin and doxorubicin-peptide) that would not function as the substrates of ABC transporters [183-185]. The final method of overcoming resistance is to administer substances inhibiting ABC transporters with anticancer drugs at the same time. These substances would reverse resistance against anticancer drugs to eventually being sensitized for anticancer drugs so they are called chemosensitizers. They are also called MDR modulators and MDR reverters. Chemosensitizers against each transporter are summarized according to the publishing years (Table 3). Of these, some are chemosensitizers against one transporter and some others against more than two transporters.

Many drugs such as the calcium channel blocker verapamil and the immunosuppressant cyclosporin A would inhibit resistance by functioning as competitive substrates of Pgp regardless of their innate pharmacological functions. Different clinical studies also showed that these drugs could reverse resistance to anticancer drugs. Verapamil is the first reported chemosensitizer inhibiting MDR [119] and its effect was also proven in the recent clinical study [186]. However, verapamil brings about cardiac toxicity at the concentration inhibiting resistance; thus, in order to resolve this problem, the attempts were made to develop (R)-verapmil [187] and verapamil analogues having lower cardiac toxicity compared with (S)-verapamil [188,189]. The immunosuppressant cyclosporin A was first reported to reverse resistance by acute leukemia against vincristine and daunorubicin [190]. Following cyclosporin A, researchers found that other immunosuppressants including FK506 and rapamycin could inhibit MDR [191]. However, when cyclosporin A is applied clinically, researchers placed efforts to develop cyclosporin analogues having few side effects due to their innate immuno suppressant effects and hepatic and renal toxicity with excellent chemosensitizing effects. As a result, PSC-833 (Valspodar), which is the non-immune suppressant analogue of cyclosporin, was developed [110]. In addition to non-immunosuppressant effect, its chemosensitivity is about 10 times higher than that by cyclosporin in Pgp-mediated MDR, so clinical studies are being performed on this drug [192]. Among those drugs having their innate pharmacological activities such as verapamil and cyclosporin A, those having chemosensitizing effect is called the first-generation chemosensitizers. The problems related with the first-generation chemosensitizers are that they generally show low effects and high toxicity at resistance-inhibiting doses. In order to supplement these problems, the chemosensitizers developed only for chemosensitizing effects are called the second-generation chemosensitizers, which include PSC-833, VX-710, LY335979, XR9051 and XR9576 [193]. Multi-national companies are pursuing the development of second-generation chemosensitizers by overcoming the problems of existing chemosensitizers (low effects, side effects, and drug-drug interaction), and some of these chemosensitizers are in the process of being tested clinically.

**Figure 4:**
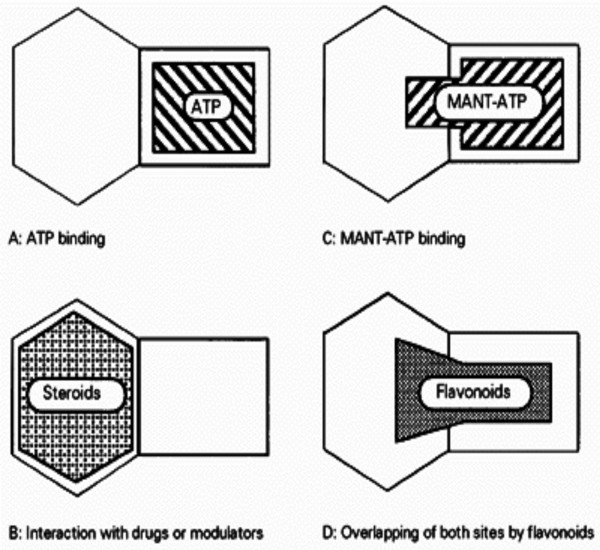
Proposed schematic model of NBDs showing the relative positions of different nucleotide- and effector-binding sites. MANT-ATP binding is prevented by preincubation with antiprogestin RU-486 and bound MANT-ATP is displaced by Ru-486, suggesting the existence of a cytosolic steroidal-interacting region adjacent to the ATP-binding site. Since the flavonoid binding is prevented by preincubation with ATP and RU-486, bound flavonoids most likely cover both ATP site and the vicinal steroid site. MANT, 2'(3')-*N*-methylanthraniloyl [3].

Most chemosensitizers bind with TMD in transporter, but steroid and flavonoid are new recently introduced chemosensitizers, which inhibit transporters by binding with NBD. The binding site of steroid is different from the binding site of ATP but is probably in the vicinity of the ATP binding site [194]. On the other hand, the flavonoid, kaempferide, is bifunctional in that it would partially block the binding of the antiprogestin RU-486 in the cytoplasm domain of Pgp and block ATP binding [195] (Fig. 4). Recently, flavonoid chemosensitizers reversing Pgp-mediated MDR have been screening. It is believed that flavonoid chemosensitizers have a significant advantage with respect with a therapeutic index (Table 4). These may be second-generation flavonoid chemosensitizers [33].

**Table 3:** Chemosensitizers inhibiting Pgp, MRP and BCRP

**Name**	**Year**	**Chemosensitizer**
Pgp	2004	**Benzyl-, phenethyl-, and alpha-naphthyl isothiocyanates **[16], diallyl sulfide [17], PK11195 [18], small scFv recombinant Pgp antibody fragment [19]
	2003	Amooranin [20], etrandrine, fangchinoline [21], ginsenoside Rg(3) [22], KR30031 [23], methylenedioxyethylamphetamine [24], protopanaxatriol ginsenosides [25], saquinavir [26], siRNA of mdr1 gene [27, 28], tRA 98006* [29]
	2002	3,5-dibenzoyl-1,4-dihydropyridines[30], PKC412 [31], pyronaridine [32], sinensetin [33]
	2001	**Agosterol A **[34], haloperidol and dihydrohaloperidol [35], SB203580 [36], tropane alkaloid esters [37], SNF4435C and D [38], tea polyphenol [39], trans-N,N'-bis(3,4-dimethoxybenzyl)-N-solanesyl-1,2-diaminocyclo hexane (N-5228) [40]
	2000	Astemizole [41], atorvastatin [42], 7-O-benzoylpyripyropene A [43], **5-O-benzoylated taxinine k [44], **clarithromycin and YM17K (3,4'-dideoxy mycaminosyl tylonolide hydrochloride) [45], cyclopamine and tomatidine[46], 3,5-diacetyl-1,4-dihydropyridines [47], 7, 8-dihydroxy-3-benzazepine [48], **doxorubicin-gallium-transferrin conjugate **[49], macrolide antibiotics (josamycin, tamolarizine) [50], nelfinavir [51] norverapamil [52], ontogen (ONT-093, formerly OC-144-093) [53], R101933 [54], taxuspine C, 2'-desacetoxyaustrospicatine and 2-desacetoxytaxinine [55], **V-104 **[56]
	1999	D-alpha-tocopheryl polyethylene glycol 1000 succinate [57], anti-MDR1 ribozymes [58], AR-2 [59], carvedilol [60], **erythromycin **[61], ketoconazole [62], kopsiflorine [63], nomegestrol [64], PAK-200S [65], **pluronic block copolymer **[66], reversin [67], ritonarvir [68], rosemary extract [69], TTD [70], XR9576(2) [71]
	1998	Ardeemins [72], AV200 [73], 5-O-benzoylated taxuspine C [74], bromocriptine [75], **dipyridamole **[76], droloxifene [77], **imidazothiazole derivatives (N276-12, N276-14, N276-17) **[78], oxalyl bis(N-phenyl)hydroxamic acid [79], tetrandine and fangchinoline [21], tiamulin [80], XR9051 [81]
	1997	**Biricodar (VX-710; Incel) **[82, 83], cyproheptadine [84]
	1996	CL 329,753 [85], indole-3-carbinol [86], **itraconazole **[87], LY335979 [88], medroxyprogesterone [89], mefloquine [90], **mifepristone (RU-486) **[91], reserpine [92]
	1995	Azelastine and flezelastine [93], B9209-005 [94], dexniguldipine (B8509-035) [95], dexverapamil [96], epidermal growth factor (EGF), insulin-like growth factor I (IGF-I) [97], quercetin [98]
	1994	**MS-209 **[99], pentoxifylline [100], Ro11-2933 (DMDP) [101], **RU486 **[102]
	1993	Dilantin [103], **GF120918**[104], meperidine, pentazocine, and methadone [105], Pgp monoclonal antibodies and antisense oligonucleotide [106], tamoxifen and toremifene [107]
	1992	Staurosporine and NA-382 [108]
	1991	Biperidil [109], SDZ PSC 833[110]
	1990	Cremophor EL [111]
	1989	Cefoperazone, cetriaxone [112], phenothiazine [113], YM534 [114]
	1987	Diltiazem[115], cyclosporine A [116]
	1986	Aamiodarone [117]
	1984	**Quinidine **[118]
	1981	Verapamil [119],
MRP	2004	**benzyl-, phenethyl-, and alpha-naphthyl isothiocyanates **[16]
	2003	**tRA 98006 **[29]
	2001	**Agosterol A **[34]
	2000	**5-O-benzoylated taxinine k **[44], 4-deacetoxyagosterol A [120], **doxorubicin-gallium-transferrin conjugate **[49], **V-104 **[56], **pluronic block copolymer **[66], quinoline-based drugs (chloroquine, quinine, **quinidine**, and primaquine) [121],
	1999	**dipyridamole **[122], **erythromycin and **ofloxacin [123], **mifepristone (RU-486) **[124], **MS-209 **[125], rifampicin [126]
	1998	**Biricodar (VX-710; Incel) **[83], **imidazothiazole derivatives (N276-12, N276-14, N276-17) **[78], NSAIDs (indomethacin, sulindac, tolmetin, acemetacin, zomepirac and mefenamic acid) [127], ONO-1078 [128], quercetin [98]
	1997	Indomethacin [129], probenecid [130]
	1996	Acrolein and chloroacetaldehyde [131], d,l-buthionine-(S,R)-sulfoximine [132], **itraconazol **[87], PAK-104P [133]
	1995	Difloxacin [134], MK571 [135]
BCRP	2004	Chrysin and biochanin A [136], genistein and naligenin [137], Imatinib mesylate (Gleevec, STI571) [138]
	2003	Estrone, diethylstilbestrol and TAG-139 [139], **tRA 98006 **[29]
	2002	Ko143 [140]
	1999	**GF120918 **[141]
	1998	fumitremorgin C [142]

* Boldface compounds indicate chemosensitizers inhibiting more than two transporters

**Table 4:** Comparison of chemosensitizing effects of flavonoids and verapamil against Pgp

**Chemosensitizer**	**IC**_**50**_^**a **^**(μM)**		**CI**^**c**^
		
	**(VCR^b^-)**	**(VCR+)**	
5,7,3',4',5' – pentamethoxyflavone	> 400	0.4	>1000
7,3',4' – trimethoxyflavone	> 400	1.2	>333.3
3',4' – dimethoxyflavone	386	1.2	321.7
3,6,3',4' – tetramethoxyflavone	> 400	1.9	>210.5
Verapamil	61	0.4	152.5
5,6,7,3',4' – pentamethoxyflavone	> 400	3.2	>125

Cytotoxic and chemosensitizing effects of chemosensitizers in the presence or absence of vincristine in Pgp-overexpressing AML-2/D100 cells.

^a^, Drug concentrations with inhibit 50% growth of the cells.

^b^, Vincristine (100 ng/ml)

^c^, Chemosensitizing index = IC_50 _(VCR-)/IC_50_(VCR+)

The fungal toxin, fumitremorgin C (FTC), is a strong inhibitor of BCRP but its use *in vivo *has been limited due to its neurotoxicity [196]. It was recently reported that the tetracyclic analogue of FTC, Ko143, is the most strong chemosensitizer aginst BCRP having little toxicity [140].

Since ABC transporters can be coexpressed in some types of cancer cells, the development of chemosensitizers against MRP and/or BCRP as well as Pgp has been highly demanding. These include VX-710 against Pgp and MRP [82,83], GF120918 against Pgp and BCRP [104,141] and tRA98006 against all three transporters [29].

## Conclusion

One of the major causes of failure in anticancer chemotherapy is resistance against anticancer drugs. Overexpression of ABC transporters such as Pgp, MRP and BCRP has been shown to be responsible for the major portion of MDR. Therefore elucidation of the structure and the function for each ABC transporter is prerequisite for understanding how these transporters work and for reversing MDR. One strategy for reversal of MDR cells expressing ABC transporters is combined use of anticancer drugs with chemosensitizers. Second-generation chemosensitizers have been developed for the purpose of obtaining higher efficacy and lower toxicity than first-generation chemosensitizers. Inhibitors of ABC transporters can be exploited to enhance the oral bioavailablilty or the brain penetration of various drugs.

Combination of a conventional anticancer chemotherapy with new strategies such as chemosensitizers, receptor-mediated targeting and nanotechnology will shed light on cancer patients in the near future.
